# Interventional Radiology in the Treatment of Musculoskeletal Tumours

**DOI:** 10.3390/curroncol33080488

**Published:** 2026-08-18

**Authors:** Sude Yarar, Panagiotis Tsagkozis

**Affiliations:** 1 Istanbul Faculty of Medicine, Istanbul University, Istanbul 34452, Turkey; sudeyarar@ogr.iu.edu.tr; 2 Department of Molecular Medicine and Surgery, Karolinska Institutet, 171 76 Stockholm, Sweden; 3 Karolinska University Hospital, 171 64 Solna, Sweden

**Keywords:** radiofrequency ablation, cryoablation, cementoplasty, high-intensity focused ultrasound, musculoskeletal

## Abstract

**Background/Objectives:** Primary benign and malignant musculoskeletal tumours are generally treated with surgical excision. Metastatic musculoskeletal disease may require both systemic and local treatment. This review focuses on the use of radiofrequency ablation (RFA), cryoablation, cementoplasty, and high-intensity focused ultrasound (HIFU) in primary musculoskeletal tumours and bone and soft-tissue metastases. **Methods:** We reviewed English-language studies addressing the indications, clinical outcomes, patient selection, and complications of these techniques in patients with primary musculoskeletal tumours or bone and soft-tissue metastases. **Results:** RFA is the preferred minimally invasive treatment for osteoid osteoma and may be considered for selected small intraosseous tumours. In symptomatic bone metastases, RFA may provide pain relief and can be combined with cementoplasty when structural support is needed. Cryoablation is used selectively for benign, locally aggressive, recurrent, and metastatic tumours, with the advantage of visualizing the ablation zone. Cementoplasty is mainly used to relieve mechanical pain and reinforce weakened bone. HIFU has been studied mainly for pain palliation in bone metastases, while evidence in primary malignant tumours remains limited. **Conclusions:** Image-guided interventions can complement surgery and radiotherapy in selected patients. Treatment choice depends on tumour type, location, treatment intent, skeletal stability, and proximity to critical structures.

## Simple Summary

Musculoskeletal tumours are usually surgically treated. However, surgery is not suitable for every patient. Image-guided treatments can provide another option, especially when an operation would carry considerable risks. Radiofrequency ablation, cryoablation, cementoplasty, and high-intensity focused ultrasound are used for different purposes. Radiofrequency ablation has an established role in treating osteoid osteoma and is also used for painful bone metastases. Cryoablation can be useful for selected benign, locally aggressive, and metastatic tumours. Cementoplasty is mainly used to relieve pain and strengthen bone weakened by tumour involvement, while high-intensity focused ultrasound has been studied mainly for pain relief in patients with bone metastases. The choice of treatment depends on the type and location of the tumour, the aim of treatment, and whether the affected bone needs additional support. Direct comparisons between these treatments could make it clearer which approach works best for different tumour types.

## 1. Introduction

Although primary musculoskeletal tumours are relatively uncommon, their management remains challenging because of their biological heterogeneity, anatomical complexity, and potential impact on function and quality of life. In contrast, metastatic involvement of the skeletal system is frequently encountered in oncologic practice and represents a major source of pain, disability, pathological fractures, spinal instability, and neurological complications. As advances in systemic therapies continue to improve cancer survival, more patients are living with skeletal metastases, creating a growing need for effective local treatment strategies that can preserve mobility and quality of life [1,2].

Surgical resection remains the standard treatment for many primary musculoskeletal tumours and represents the cornerstone of local disease control. However, extensive resections may be associated with significant morbidity, prolonged recovery, and functional impairment, particularly when tumours are located near critical neurovascular structures or within anatomically complex regions [3]. Similarly, although external-beam radiotherapy remains an established modality for pain palliation and local control in skeletal metastases, its effects may be delayed, repeated treatment can be restricted by cumulative dose constraints, and additional stabilization may be required when structural compromise is present [4]. These limitations have stimulated interest in less invasive approaches capable of providing tumour control, pain relief, and structural support while minimizing treatment-related morbidity [5].

The rapid development of musculoskeletal interventional oncology has transformed the role of radiology in tumour care. Once largely confined to diagnostic imaging and image-guided biopsy, radiology now plays an active therapeutic role through minimally invasive procedures designed to achieve tumour destruction, pain palliation, and skeletal stabilization [1,6]. Advances in cross-sectional imaging, navigation technologies, ablation systems, and thermal protection techniques have enabled increasingly precise treatment of musculoskeletal lesions, including those near vulnerable neural, cutaneous, visceral, or articular structures [7,8,9].

Consequently, image-guided interventions have become an increasingly important component of multidisciplinary musculoskeletal oncology. These procedures may be used with curative intent in selected benign and oligometastatic lesions or integrated into palliative treatment strategies aimed at reducing pain, preserving mobility, and maintaining skeletal integrity [2,4]. Their value is greatest when treatment goals are clearly defined and when patient selection is guided by multidisciplinary discussion [6,10].

The decision to use an image-guided procedure depends on more than technical feasibility. Lesion size and soft-tissue extension are important, as well as proximity to nerves or other critical structures. Previous radiotherapy, performance status, and expected survival may also influence treatment. Bone stability becomes especially important in the spine and long bones. In spinal metastases, the Spinal Instability Neoplastic Score (SINS) can help assess this. Scores of 0–6 are considered stable, 7–12 are considered potentially unstable, and 13–18 are considered unstable, with spinal assessment generally recommended for scores of 7 or higher [11]. Fracture risk should also be considered in long-bone metastases, particularly for lytic lesions in weight-bearing bones. Mirels’ score is commonly used, although it has limitations and is best considered together with the clinical and imaging findings. Cortical involvement, lesion size and location, pain, functional status, and overall prognosis may ultimately affect whether local treatment alone is appropriate or additional stabilization is needed [12,13].

A range of interventional techniques is used in musculoskeletal oncology, including thermal ablation, cement augmentation, embolization, and percutaneous stabilization procedures. This review focuses on radiofrequency ablation, cryoablation, cementoplasty, and high-intensity focused ultrasound because, together, they address the main interventional goals of tumour ablation, pain relief, and skeletal stabilization. Other techniques are not discussed as separate treatment modalities, but are included when relevant to combined treatment or skeletal stabilization, particularly microwave ablation, vertebral augmentation, osteoplasty, and fixation techniques [5,14].

## 2. Methods

This narrative review summarizes the current clinical use of radiofrequency ablation (RFA), cryoablation, cementoplasty, and high-intensity focused ultrasound (HIFU) in the management of primary and secondary musculoskeletal tumours. The literature search was carried out in PubMed and Google Scholar between 25 March and 7 June 2026. Search terms included the terms “musculoskeletal tumour,” “bone tumour,” “soft-tissue tumour,” “bone metastasis,” “radiofrequency ablation,” “RFA,” “cryoablation,” “cementoplasty,” “high-intensity focused ultrasound,” “HIFU,” “MR-guided focused ultrasound,” and “MRgFUS.” Google Scholar results were screened based on the relevance of titles and abstracts, and full texts were reviewed when necessary according to the same selection criteria used for PubMed.

English-language studies were considered if they addressed the clinical use, outcomes, patient selection, or complications of these four techniques. These included prospective and retrospective studies, clinical trials, case series, systematic and narrative reviews, technical or educational reviews, and selected consensus or guidance documents. Studies focusing on other interventional techniques, such as microwave ablation, embolization, electrochemotherapy, neurolysis, or percutaneous fixation, were generally not included unless they provided relevant comparative or supporting information.

Study selection was performed by the first author and confirmed by the last author. Studies were selected based on their relevance to the scope of the review, particularly those reporting clinical indications, technical or clinical outcomes, pain response, local tumour control, complications, and patient selection. Reference lists of relevant review articles were also checked to identify additional studies of clinical or historical importance. As this was a narrative review, no formal risk-of-bias assessment or quantitative evidence synthesis was performed. In total, 116 references were included in the final review.

The studies did not always define outcomes in the same way. Technical success generally meant that the planned treatment was completed successfully, although the exact criteria varied between studies. Pain response was measured using different pain scales and response criteria, while local tumour control was assessed using the imaging and follow-up criteria reported in each study. Complications were also reported differently across studies. These differences in definitions, follow-up, and reporting were considered when reviewing the results, rather than comparing the reported outcomes directly.

## 3. Radiofrequency Ablation

### 3.1. Technique, Advantages, and Limitations

Radiofrequency ablation (RFA) is one of the earliest and most extensively studied thermal ablation techniques in musculoskeletal interventional oncology. Initially established for the treatment of osteoid osteoma, its applications have expanded to selected benign bone tumours, painful skeletal metastases, spinal and extraspinal metastatic lesions, and carefully selected cases in which local tumour control is technically feasible [15,16].

RFA involves placing an electrode within the target lesion under image guidance and applying an alternating electrical current. This generates heat within the tissue, resulting in coagulative necrosis. CT is commonly used to guide the procedure in bone lesions, as it provides clear visualization of the lesion and surrounding anatomy during electrode placement. The temperature and duration of treatment vary depending on the device used, lesion size, and anatomical location, although temperatures of approximately 80–95 °C are commonly reported. For malignant lesions treated with local-control intent, the ablation zone should ideally extend beyond the visible tumour margins, whereas in benign lesions such as osteoid osteoma, treatment is generally directed at the nidus itself [1,15].

Probe configuration is an important technical consideration, particularly in larger or irregularly shaped lesions. Small lesions, such as osteoid osteoma, can often be treated with a single probe [17]. In contrast, larger lesions may require repeated probe repositioning, overlapping ablations, multiple probe placements, internally cooled electrodes, expandable multi-tined electrodes, or cluster systems to achieve adequate tumour coverage. Increasing the ablation volume, however, also increases the need for careful thermal monitoring and protection of adjacent neural, articular, cutaneous, or visceral structures [15,18,19].

RFA remains widely used because it is minimally invasive, reproducible, broadly available, relatively inexpensive, and supported by extensive clinical experience [18]. It can also be repeated when necessary and combined with other local treatments, including cementoplasty, vertebral augmentation, radiotherapy, or systemic therapy [20,21].

Important limitations should be recognized. Procedural success may be influenced by lesion size, tissue composition, local anatomy, and adjacent vascular structures. The heat-sink effect may reduce thermal injury in highly vascular environments, while large or irregular lesions may be difficult to treat completely when safe margins cannot be achieved. Unlike cryoablation, RFA does not allow direct visual monitoring of the entire ablation zone during treatment. Therefore, safety and completeness depend heavily on pre-procedural imaging, expected thermal spread, probe positioning, and operator experience. Lesions adjacent to major nerves, articular cartilage, skin, bowel, or the spinal cord require meticulous planning, and thermal protection techniques such as hydrodissection, gas dissection, temperature monitoring, or deliberate reduction of the planned ablation zone may be necessary [8,20].

Although major complications are uncommon, reported adverse events include bleeding, infection, skin burns, pathological fractures, and injury to adjacent neurovascular structures. In metastatic bone disease, RFA can reduce tumour burden and pain, but it does not restore mechanical stability. For this reason, structurally compromised or weight-bearing lesions often require cementoplasty, vertebral augmentation, osteoplasty, fixation, or surgery [20,21].

### 3.2. Benign Bone Tumours

The strongest evidence supporting RFA in musculoskeletal oncology comes from osteoid osteoma. Historically, surgical excision was considered the standard treatment, but accurate localization of the nidus often required removal of surrounding bone and could be associated with prolonged recovery or structural morbidity. CT-guided RFA changed the management of osteoid osteoma by allowing direct, minimally invasive treatment of the nidus with rapid pain relief and limited damage to surrounding tissues [17,22].

RFA has shown consistently good outcomes in the treatment of osteoid osteoma. In a series of 47 patients, Woertler et al. reported clinical success after the first procedure in 44 patients (94%). Three patients developed recurrent pain during follow-up and were successfully treated with a second ablation, resulting in a secondary success rate of 100%. No immediate or delayed complications were reported during a mean follow-up of 22 months [23]. Cioni et al. reported primary clinical success in 30 of 38 patients (78.9%), with a secondary success rate of 97.2% after repeat treatment. Thirty patients experienced pain relief and returned to normal activities within one week. The mean follow-up was 35.5 months, and no late recurrences were reported after successful treatment [15,23,24].

RFA has also been used in selected patients with chondroblastoma, although the evidence is limited to small case reports and case series. It may be useful for lesions in epiphyseal or apophyseal locations, where surgery can risk damage to the adjacent joint or growth plate. In a series of four patients, Tins et al. reported no tumour recurrence during follow-up, although two patients developed complications [25]. Other small reports have also described pain relief and favourable imaging findings after RFA, including in lesions of the femoral head [26,27,28]. However, the small number of patients studied makes it difficult to draw firm conclusions about long-term tumour control and safety.

For larger or biologically more complex bone tumours, the available evidence remains limited but suggests a possible role in carefully selected patients. Small prospective and retrospective experiences have reported encouraging local control and pain reduction, but these applications should not be interpreted as a uniform replacement for surgery. Overall, RFA is best established for osteoid osteoma, while its role in other benign or intermediate lesions remains selective and should be guided by multidisciplinary assessment, technical feasibility, and proximity to articular, neural, or soft-tissue structures [5,19].

### 3.3. Metastatic Disease

RFA has become an important option in the management of metastatic musculoskeletal disease, particularly for patients with localized painful skeletal metastases. Although radiotherapy remains a cornerstone of treatment, pain relief may be delayed, incomplete, or limited by prior radiation exposure. In this setting, percutaneous ablation offers a repeatable local treatment that can provide relatively rapid palliation and can be integrated with systemic therapy, radiotherapy, or other local interventions [20,29].

The need for biopsy before RFA or cryoablation depends on the clinical setting. When bone metastasis is already supported by the patient’s cancer history and imaging findings, biopsy is not routinely needed before treatment. It may be appropriate when the primary tumour is unknown, when more than one primary malignancy is possible, or when tissue sampling could guide systemic treatment [1].

Pain relief after RFA is thought to result from both tumour destruction and an effect on the tumour–bone interface. Clinical studies have consistently shown improvement in pain after treatment. In a multicentre study of 43 patients, Goetz et al. reported a decrease in mean worst pain from 7.9 at baseline to 4.5 at four weeks and 1.4 at 24 weeks, with clinically significant pain relief in 95% of patients [30]. Dupuy et al. also found significant pain reduction at one and three months in a prospective multicentre study of 55 patients [31]. More recently, the OPuS One study of 100 patients showed a decrease in mean worst pain from 8.2 at baseline to 3.5 at six months, with four treatment-related adverse events [32]. Together with additional prospective data, these findings support RFA as a palliative option for selected patients with painful bone metastases [30,31,32,33].

Because RFA does not provide mechanical stability, it is often combined with cementoplasty or vertebral augmentation in osteolytic or structurally compromised lesions. RFA addresses tumour-related pain and local tumour burden, while cement augmentation provides additional support to weakened bone. Hoffmann et al. treated 22 patients with 28 painful bone metastases using RFA followed by osteoplasty. Mean pain scores decreased from 8.5 before treatment to 5.5 on the first day and 3.5 at six months, with no major complications reported [21]. In a prospective study of 30 patients, Fares et al. found significant reductions in pain, morphine use, and disability during 12 weeks of follow-up [34]. Pusceddu et al. reported similar findings in 17 patients treated with steerable RFA and cementoplasty, with median pain scores decreasing from 7 before treatment to 2 at one month and 0 at 12 months. Two cases of cement leakage were reported, and no local recurrence was observed during the 12-month follow-up [35]. These findings suggest that combining RFA with cement augmentation may be useful when both pain control and mechanical support are needed.

Beyond pain palliation, thermal ablation has also been used for local tumour control in selected patients with limited metastatic bone disease. The evidence in this setting is more difficult to interpret, as several studies include both RFA and cryoablation rather than evaluating RFA alone. Cazzato et al. studied 46 patients with 49 bone metastases and reported local progression-free survival rates of 76.8% at one year and 71.7% at two years. However, only 12 of the 49 lesions were treated with RFA, with the remainder treated with cryoablation [36]. In spinal metastases, Wallace et al. reported radiographic local control rates of 89% at three months, 74% at six months, and 70% at one year after RFA combined with vertebral augmentation [37]. Other studies have also reported local control after thermal ablation, but differences in tumour type, anatomical site, treatment technique, and follow-up make direct comparisons difficult [38,39]. For this reason, the evidence for durable local control with RFA alone remains limited, and its use for this purpose should be considered on a case-by-case basis within a multidisciplinary treatment plan.

Overall, RFA is a flexible and clinically useful technique in musculoskeletal oncology. Its clearest roles are curative treatment of osteoid osteoma and pain palliation for localized skeletal metastases. Its use in other benign tumours and local control settings is more selective, requiring careful assessment of lesion size, location, expected ablation margins, structural stability, and overall oncologic context. The principal clinical applications, advantages, and limitations of RFA in musculoskeletal oncology are summarized in Table 1.

## 4. Cryoablation

### 4.1. Technique, Advantages, and Limitations

Cryoablation is a minimally invasive thermal ablation technique that destroys tumour tissue through controlled freezing. Modern percutaneous cryoablation developed from earlier open cryosurgical techniques and has become increasingly relevant in musculoskeletal oncology because it allows for treatment of both bone and soft-tissue lesions under image guidance [40,41]. Its main applications include painful skeletal metastases, selected oligometastatic lesions, desmoid tumours, osteoid osteoma, and selected recurrent or unresectable malignant bone and soft-tissue tumours [1].

Cryoablation uses rapid gas expansion within the cryoprobe to generate the low temperatures needed for tissue ablation. Freezing damages tumour cells through ice-crystal formation and also affects the local microcirculation, with apoptosis contributing to cell death at the periphery of the treated area. Repeated freeze–thaw cycles are generally used to increase tissue destruction. CT is the most commonly used imaging method for musculoskeletal cryoablation as it allows both probe placement and monitoring of the developing ice ball. MRI and ultrasound can also be used in selected cases [41].

A major advantage of cryoablation is that the ablation zone can be monitored during treatment. The visible ice ball helps the operator assess tumour coverage and avoid adjacent vulnerable structures [42]. However, the visible margin corresponds to non-lethal temperatures and therefore must extend beyond the tumour margin to achieve complete treatment. Multiple cryoprobes can be used simultaneously to create large, overlapping, and irregular ablation zones, making cryoablation particularly useful for large or geometrically complex musculoskeletal lesions [40,43].

Cryoablation is generally well tolerated in palliative musculoskeletal oncology and has been associated with clinically meaningful pain reduction in patients with painful metastatic disease [44]. It can also be repeated and combined with cementoplasty, vertebral augmentation, embolization, or surgery when necessary. However, limitations include longer procedure durations, higher equipment costs, potential difficulty in monitoring the ice ball in sclerotic bone or fat, and the need for meticulous protection near nerves, bowel, skin, articular cartilage, and the spinal canal. Protective techniques include hydrodissection, gas dissection, temperature monitoring, neuromonitoring, and physical displacement of critical structures [8].

Direct comparisons of RFA and cryoablation are uncommon. In one retrospective study, 274 patients with 367 bone metastases were treated with either RFA or cryoablation. RFA was used for 66 metastases and cryoablation for 301. Major complications occurred in 1.5% and 2.7% of procedures, respectively, with no significant difference between the two techniques. Minor complications were reported more often after RFA (33.3% vs. 6.0%). Most of this difference came from post-procedural pain, which was reported after 30.3% of RFA procedures and 2.3% of cryoablation procedures. After propensity score matching, minor complications were still more frequent with RFA, while major complication rates remained similar [45]. The complications reported in selected clinical studies of RFA and cryoablation are summarized in Table 2.

### 4.2. Benign Bone and Soft-Tissue Tumours

Among benign bone tumours, osteoid osteoma is one of the better studied indications for cryoablation. Although RFA remains the more established percutaneous treatment, cryoablation provides an alternative in selected cases, particularly when visualization of the ablation zone is useful during treatment. In an early paediatric series of six patients, Wu et al. achieved technical and clinical success in all cases, with mean VAS scores decreasing from 6.57 before treatment to 0.57 at one month and no recurrence during a mean follow-up of 28.7 months [50]. Meng et al. later reported similar findings in 15 patients, with mean VAS scores decreasing from 6.33 before treatment to 0.73 within seven days and 0.13 after more than 12 months. No recurrence was observed during a median follow-up of 17 months [51]. In a larger series of 50 patients, Le Corroller et al. reported clinical success in 48 (96%), with median VAS scores decreasing from 8 before treatment to 0 at six weeks and remaining at 0 during longer-term follow-up. One recurrence occurred at 11 months and was successfully treated with repeat cryoablation. Three minor complications were reported, with no major complications [52].

Cryoablation has also been explored in other benign or intermediate bone lesions, including osteoblastoma, chondroblastoma, aneurysmal bone cysts, symptomatic bone cysts, and selected tumour-like lesions. The evidence for these indications is more limited and mainly based on small series or case reports. Therefore, cryoablation should be considered selectively, especially when surgery would be technically difficult, functionally morbid, or disproportionate to the biological behaviour of the lesion [40,41].

Desmoid tumours are an important soft-tissue indication for cryoablation, although treatment is not usually required at diagnosis. Active surveillance is generally the initial approach, with intervention considered when the tumour progresses or causes persistent symptoms or functional impairment [53,54]. In the prospective multicentre CRYODESMO-01 study, 50 patients with progressive desmoid tumours underwent cryoablation. At 12 months, 86% had no disease progression, alongside improvements in pain and functional status [55]. A recent review reported an average pain reduction of 79% and tumour-volume reduction of 71.5% across studies [56]. Cryoablation can therefore be considered for selected patients who need local treatment, particularly when surgery would be difficult or carry substantial morbidity [53,54].

### 4.3. Primary and Recurrent Malignant Bone and Soft-Tissue Tumours

The role of cryoablation in primary malignant musculoskeletal tumours is more limited than in benign disease or metastases. Surgery, often combined with radiotherapy and systemic therapy, remains the standard treatment for most primary bone and soft-tissue sarcomas. Cryoablation should therefore not be presented as a replacement for established oncologic treatment, but rather as an adjunctive or alternative local treatment in carefully selected patients who are not suitable candidates for surgery, have recurrent disease, have exhausted radiotherapy options, or require local control of limited treatment-resistant disease [41].

Early reports of cryoablation for recurrent or metastatic bone and soft-tissue tumours involved relatively small patient groups. Susa et al. treated 11 lesions in nine patients who were not suitable for surgery, chemotherapy, or radiotherapy. No local recurrence was observed during a median follow-up of 24.1 months, although three procedure-related complications occurred [57]. Given the small and heterogeneous cohort, these findings should be interpreted cautiously. Cryoablation has also been studied in recurrent retroperitoneal soft-tissue sarcoma and sacral chordoma, while Lippa et al. examined criteria for selecting patients with recurrent soft-tissue sarcoma for treatment [58,59,60].

A larger retrospective study by Pal et al. included 141 patients with 250 recurrent or metastatic soft-tissue sarcoma lesions. Local progression-free survival was 86% at one year and 80% at two years, with an overall complication rate of 2%. Inadequate ice-ball coverage was associated with a higher risk of local progression [49]. Although these results are promising, the evidence remains largely retrospective. For now, cryoablation is mainly an option for selected patients with recurrent or metastatic disease and does not replace surgical resection.

Thus, in malignant musculoskeletal tumours, cryoablation is best framed as a selective local therapy rather than a general curative substitute. Its strongest rationale is in recurrent, unresectable, oligometastatic, or treatment-refractory disease where local control may reduce symptoms, delay progression, or allow continuation of systemic therapy.

### 4.4. Metastatic Disease

The strongest evidence supporting cryoablation in musculoskeletal oncology comes from metastatic disease, particularly painful skeletal metastases. Bone metastases are a major cause of cancer-related pain, disability, pathological fracture, and impaired quality of life. Although radiotherapy remains a cornerstone of treatment, pain relief may be delayed, incomplete, or limited by prior radiation exposure. In this setting, cryoablation offers a repeatable, image-guided local treatment that can provide rapid palliation and can be integrated with systemic therapy or previous radiotherapy [61].

Pain reduction after cryoablation has been consistently reported in prospective multicentre studies. Callstrom et al. treated 61 patients with painful bone metastases, with mean worst pain decreasing from 7.1 at baseline to 5.1 at one week, 4.0 at four weeks, and 1.4 at 24 weeks. One major complication (2%) was reported [47]. In the MOTION study, which included 66 patients, worst pain decreased by a mean of 2.61 points at eight weeks and continued to improve during follow-up. Three serious adverse events were reported [48]. Cryoablation may therefore provide sustained pain relief for patients with painful bone metastases, particularly when standard treatment has not provided adequate relief.

Local tumour control is another potential role for cryoablation in patients with limited metastatic disease. McMenomy et al. treated 52 oligometastatic lesions in 40 patients and achieved local control in 45 lesions (87%) over a median follow-up of 21 months. Control differed between bone and soft-tissue metastases (68% and 97%, respectively) [62]. Similar experience has been reported in vertebral metastases, where Tomasian et al. achieved local control in 30 of 31 lesions (96.7%) over a median follow-up of 10 months [63,64]. Cryoablation has also been evaluated in bone metastases from renal cell carcinoma [65]. While these results are promising, the studies vary in tumour type, treatment intent, anatomical site, and length of follow-up. Long-term local control is therefore less well established than pain palliation.

In structurally vulnerable bone, cryoablation can be combined with cementoplasty, particularly for lytic lesions in weight-bearing areas [66]. Cementoplasty helps stabilize the weakened bone while cryoablation is used to treat the tumour and associated pain. Coupal et al. treated 48 patients with large, painful pelvic bone metastases who were not candidates for surgery. All procedures were technically successful, and mean pain scores decreased from 7.9 before treatment to 1.2 at 24 h, with pain relief maintained during short-term follow-up [67]. Combining the two techniques can therefore be useful when a painful lesion has also weakened the surrounding bone.

Overall, cryoablation is a valuable technique for the management of metastatic musculoskeletal disease. Its clearest role is pain palliation in localized skeletal metastases, especially when radiotherapy is insufficient, contraindicated, or has been exhausted. Its role in local control is promising in selected oligometastatic disease, while its use in structurally compromised bone should usually be combined with cementoplasty, vertebral augmentation, fixation, or surgery rather than used as an isolated treatment. The principal clinical applications, advantages, and limitations of cryoablation in musculoskeletal oncology are summarized in Table 3.

## 5. Cementoplasty

### 5.1. Technique, Advantages, and Limitations

Cementoplasty is a minimally invasive image-guided stabilization technique based on the percutaneous injection of polymethyl methacrylate (PMMA) into bone weakened by tumour-related osteolysis. Initially developed in the spine as vertebroplasty and kyphoplasty, cement augmentation has gradually expanded to extraspinal sites, including the sacrum, pelvis, acetabulum, and selected long bones [68]. Its primary role in musculoskeletal oncology is mechanical stabilization and pain palliation rather than direct tumour eradication.

The therapeutic effect of cementoplasty is mainly mechanical. Osteolytic destruction weakens cancellous bone and may produce painful micromotion at the tumour–bone interface. By filling the defect, PMMA reinforces weakened bone, improves load distribution, and may reduce mechanical pain [69]. The exothermic polymerization reaction and local chemical effects may also contribute to analgesia, but the main clinical benefit remains stabilization rather than antitumour activity. Therefore, cementoplasty should be regarded primarily as an analgesic and structural procedure; when local tumour control is required, it is often combined with thermal ablation or other oncologic treatments [68].

The procedure may be performed under fluoroscopy, CT, cone-beam CT, or combined imaging guidance. CT is useful for assessing cortical destruction, extraosseous extension, and safe needle trajectories, whereas fluoroscopy allows for real-time monitoring of cement injection and early recognition of leakage. Careful preprocedural imaging is essential, particularly in vertebral, sacral, acetabular, and pelvic lesions where cement leakage into the spinal canal, neural foramina, joints, veins, or adjacent soft tissues may have clinical consequences [70].

Several features have supported the widespread use of cementoplasty in patients with advanced malignancy. It is minimally invasive, can often be performed under conscious sedation, provides rapid pain relief, and may improve mobility with shorter recovery times than surgical stabilization [71]. These advantages are especially valuable in patients with limited life expectancy, poor surgical candidacy, or persistent mechanical pain despite radiotherapy, systemic therapy, or analgesics.

Important limitations should also be recognized. Cementoplasty provides limited direct antitumour effects and does not replace surgery in patients with major instability, spinal cord compression, or grossly displaced pathological fractures. Cement leakage is the most frequent complication, although most leakage events are asymptomatic. When leakage occurs into critical areas such as the spinal canal or neural foramina, clinically significant neural compromise may occur. Biomechanical limitations are also important: cement augmentation is most effective in regions subjected predominantly to compressive loading, such as vertebral bodies, the sacrum, and the acetabulum. In long bones, where bending, shear, and torsional forces are substantial, cement alone may be insufficient and fixation or reinforced osteoplasty may be required [72,73].

Calcium phosphate cement (CPC) is another material that can be used to fill bone defects after musculoskeletal tumour surgery. Matsumine et al. described 56 patients treated with CPC after tumour curettage or resection. At final follow-up, radiographic adaptation to the surrounding bone was good or excellent in all patients, and all had returned to full physical function. Two patients who received larger amounts of CPC developed non-infectious discharge and local inflammation. CPC also has limited strength against torsional forces, which is important when it is used in large defects of long bones. In these cases, additional fixation may be necessary. Its use in this setting is mainly for filling bone defects after tumour surgery and differs from percutaneous PMMA cementoplasty [74].

### 5.2. Clinical Applications

The earliest and most extensive cement augmentation literature comes from vertebroplasty and kyphoplasty for painful malignant vertebral compression fractures. In appropriately selected patients without significant spinal cord compression or neurological deficit, vertebral augmentation can provide rapid pain relief, improve function, and stabilize vertebral bodies weakened by metastatic disease or myeloma [75,76,77]. In Weill et al., clear or moderate pain improvement was seen after 31 of 33 evaluable procedures, and 73% of patients continued to report improvement at six months [75]. Fourney et al. reported marked or complete pain relief after 84% of evaluable procedures, with a significant reduction in pain still present at one year [77]. However, posterior wall destruction, epidural extension, severe spinal instability, active infection, coagulopathy, and asymptomatic compression fractures require careful evaluation and may represent contraindications or indications for alternative treatment [78].

In prospective and retrospective extraspinal cementoplasty studies, patients experienced rapid reductions in pain scores, reduced analgesic requirements, and improved mobility, particularly when lesions involved the pelvis, acetabulum, or other weight-bearing regions where mechanical pain was prominent. Botton et al. reported complete or partial pain relief after 88% of 42 procedures. Of the 22 patients who could not walk before treatment, 16 (73%) were able to walk again after cementoplasty [79]. In one prospective series of extraspinal painful lytic lesions, feasibility was 100%, pain relief was rapid and sustained, and mobility improved in a substantial proportion of patients with lower limb or pelvic lesions [71]. Another prospective series reported marked visual analogue scale (VAS) reduction and suspension of narcotic drugs in 47 of 50 patients, although delayed fractures in two femoral diaphyseal lesions emphasized the limitation of cementoplasty alone in long weight-bearing bones [72].

Pelvic and acetabular lesions represent particularly important indications because pain in these sites directly affects sitting, standing, gait, and independence. Acetabuloplasty may improve load transmission across the hip joint when the acetabular roof or columns are involved, while sacroplasty may reduce pain and improve sitting or ambulation in selected sacral lesions. However, complex anatomy, neural foramina, joint proximity, and cortical destruction require careful planning, and extensive structural loss may require screws, osteosynthesis, or surgery rather than cement alone [68].

In long bones, cementoplasty should be used more selectively. PMMA may help reduce pain and provide some local reinforcement, but it does not reliably resist torsional and bending forces. Therefore, lesions in the femoral diaphysis, subtrochanteric region, humerus, or other mechanically demanding sites often require adjunctive fixation, screw-assisted cementoplasty, intramedullary nailing, or surgical stabilization [72,80]. Experimental work has looked at whether adding reinforcement could improve stability in these lesions. In the study by Koirala and McLennan, reinforcement increased mechanical strength to some extent, but the difference from cementoplasty alone was not statistically significant and both approaches remained considerably weaker than intact bone [80]. This is an important limitation when cementoplasty is considered for long bones that are exposed to substantial mechanical stress.

### 5.3. Combined Ablation and Cementoplasty

The integration of cementoplasty with thermal ablation has become an important development in musculoskeletal interventional oncology. This combined approach is based on the complementary effects of the two techniques: ablation targets tumour-related pain and local tumour burden, whereas cementoplasty addresses mechanical pain and structural weakness. This distinction is clinically important because thermal ablation alone does not restore skeletal strength and may further weaken already compromised bone [81].

Early experience with combined radiofrequency ablation and cementoplasty showed that the approach was technically feasible and associated with significant pain reduction in painful neoplastic bone lesions involving the spine, acetabulum, sacrum, pubis, and humerus [21,81]. Lane et al. treated 53 lesions with technical success in all cases, with mean pain scores falling from 7.2 before treatment to 3.4 afterwards [81]. Tian et al. reported a similar pattern in extraspinal bone metastases, where mean VAS scores decreased from 7.1 at baseline to 2.2 within 24 h and 1.3 at six months [82].

Microwave ablation combined with cementoplasty has also been used for painful spinal metastases at risk of structural compromise. In this approach, microwave ablation targets tumour-related pain and local tumour burden, while cementoplasty reinforces osteolytic bone and addresses the mechanical component of pain. Early experience showed effective palliation in selected spinal metastases, including cases treated with additional cement augmentation [83]. In a series of 69 patients, median pain scores decreased from 7.0 before treatment to 2.0 at 2–4 weeks and remained at 2.0 at 20–24 weeks [84]. In patients with recurrent spinal metastases after radiotherapy, mean VAS scores fell from 6.3 before treatment to 1.7 at one month and 1.3 at six months [85].

Similar combined stabilization strategies have also been reported with cryoablation, particularly in large pelvic or axial-loading skeletal metastases where both tumour-related pain and mechanical vulnerability contribute to symptoms [66,67].

The clinical literature supports cementoplasty as a central palliative stabilization technique in musculoskeletal oncology. Its clearest role is rapid pain relief and mechanical reinforcement in painful osteolytic lesions, particularly in the spine, pelvis, sacrum, and acetabulum. In long bones or severely unstable lesions, cementoplasty alone is often insufficient and should be combined with fixation or replaced by surgery when appropriate. When tumour-related pain or local tumour burden is prominent, combined ablation and cementoplasty offers a rational multimodal strategy that addresses both biological and mechanical components of metastatic bone disease. The main clinical applications, expected benefits, limitations, and adjunctive procedures associated with cementoplasty are summarized in Table 4.

## 6. High-Intensity Focused Ultrasound

### 6.1. Technique, Advantages, and Limitations

High-intensity focused ultrasound (HIFU) is a non-invasive ablation technique, most often used in musculoskeletal oncology with magnetic resonance guidance (MRgFUS). It works by focusing ultrasound energy on a defined target, where the resulting heat causes coagulative necrosis. MRI helps with treatment planning and localization of the lesion and also allows temperature changes to be monitored during treatment. One practical advantage of HIFU is that the procedure does not require a needle or probe to pass through the skin. This distinguishes it from radiofrequency ablation, microwave ablation, and cryoablation and avoids complications related to probe insertion, such as tract bleeding, infection, or soft-tissue injury [86,87].

The biological effect of HIFU in bone differs from soft-tissue ablation. Because cortical bone absorbs ultrasound energy efficiently, treatment of painful bone lesions often relies on heating at the cortical surface and adjacent periosteal tissues rather than deep penetration through mineralized bone. This mechanism is particularly relevant in painful skeletal metastases, where periosteal denervation and thermal injury at the tumour–bone interface are thought to contribute substantially to analgesia. When a lesion has a large extraosseous or transcortical soft-tissue component, the ultrasound beam may also be directed toward the soft-tissue tumour component in a manner more similar to soft-tissue ablation [87,88,89].

Several features make HIFU attractive in musculoskeletal oncology. It is completely non-invasive, does not require ionizing radiation, can be repeated when necessary, and may be integrated with radiotherapy or systemic treatment. MR guidance also permits thermal dose monitoring and adjustment during the procedure, which is an important safety advantage. These characteristics are particularly valuable in patients who have already received radiotherapy, in young patients with benign lesions such as osteoid osteoma, and in patients for whom percutaneous access is difficult or undesirable [90,91,92].

Despite these advantages, HIFU is not suitable for all musculoskeletal lesions. A safe acoustic window is required, and treatment may be limited by intervening bowel, lung, neurovascular structures, surgical hardware, scar tissue, or unfavourable lesion geometry [93]. Lesions located deep in the pelvis, near critical nerves, or behind highly reflective cortical bone may be technically challenging. In addition, because HIFU does not provide mechanical reinforcement, patients with pathological fractures, major structural instability, or spinal cord compression require careful multidisciplinary assessment and may be better managed with stabilization, cementoplasty, surgery, or other combined approaches. Procedure duration, patient positioning, pain during sonication, and the need for careful thermal monitoring may also limit its use in frail patients or in lesions requiring extensive treatment volumes.

Magnetic resonance (MR) thermometry is another important but imperfect component of treatment. Although it allows real-time monitoring of temperature changes in adjacent soft tissues, cortical bone itself provides weak MR signals so temperature elevation within bone cannot be directly measured reliably. Respiratory motion, patient movement, field inhomogeneity, and arterial ghosting may also degrade thermometry quality. These limitations emphasize the need for careful pre-procedural planning, patient positioning, sedation or anaesthesia planning, and post-treatment assessment [92,94].

Therefore, HIFU is best suited to technically accessible lesions where the primary goal is pain palliation or local thermal treatment rather than mechanical support. In unstable lesions, impending pathological fractures, or lesions requiring structural reinforcement, cementoplasty, osteosynthesis, surgical fixation, or another stabilization strategy may be required.

### 6.2. Benign Bone and Soft-Tissue Tumours

Among benign bone tumours, osteoid osteoma is the most relevant indication for HIFU. The appeal of MRgFUS in osteoid osteoma lies in its non-invasive and radiation-free nature, which is particularly important in children, adolescents, and young adults. Although radiofrequency ablation remains the most established minimally invasive treatment, MRgFUS has shown promising results in selected, technically targetable osteoid osteomas [95,96].

In a small paediatric study, eight of nine patients treated with MR-HIFU were pain-free four weeks after treatment, with the median VAS score falling from 6 to 0. All nine patients in the RFA group were also pain-free, and no serious adverse events were seen with MR-HIFU [97]. Masciocchi et al. reported comparable results: treatment was successful in 14 of 15 patients (93.3%) with MRgFUS and in all 15 patients treated with RFA [96]. However, eligibility was selective; lesions close to the skin, physis, major nerves, or spine, and deeply intramedullary lesions may be less suitable [93].

Desmoid-type fibromatosis has also been treated with HIFU, especially in cases where surgery may have led to functional or cosmetic problems. Many desmoid tumours can initially be followed without treatment as some remain stable or regress over time. Treatment is more often considered when the tumour continues to grow, symptoms become more troublesome, or its location raises concern about future functional problems [98]. In the multicentre study by Düx et al., which included 105 patients, median tumour volume fell from 114 mL to 51 mL during follow-up. Pain also improved, with scores decreasing from 6 to 3 [99]. Zhang et al. studied 111 patients and found a median non-perfused volume rate of 84.9%, together with a 36.1% reduction in tumour volume at three months [100]. HIFU is not suitable for every desmoid tumour, however. Large lesions and tumours close to the skin or major nerves can be more difficult to treat, and a suitable acoustic window is needed.

Overall, HIFU has a promising but selective role in benign musculoskeletal disease. It should be considered mainly when the lesion is technically targetable and when the non-invasive nature of treatment provides a meaningful advantage over established percutaneous or surgical approaches.

### 6.3. Primary and Recurrent Malignant Bone and Soft-Tissue Tumours

The role of HIFU in primary malignant musculoskeletal tumours remains much less established than its role in painful bone metastases. Surgery, often combined with chemotherapy and/or radiotherapy, remains the standard treatment for most primary bone and soft-tissue sarcomas. HIFU should therefore not be presented as a replacement for oncologic surgery or standard multimodal therapy. Its potential role is best framed as an adjunctive or alternative local treatment in highly selected patients with unresectable, recurrent, functionally morbid, or medically inoperable disease [101].

The primary bone tumour literature includes selected HIFU series in osteosarcoma and other malignant bone tumours. Yu et al. treated 27 patients with locally unresectable recurrent osteosarcoma. Fourteen patients (51.8%) responded to treatment and local disease control was achieved in 23 (85.2%). Median local progression-free survival was 14 months and median overall survival was 21 months [102]. In a larger series of 80 patients with primary malignant bone tumours, complete ablation on imaging was reported in 69 patients, with a five-year overall survival rate of 50.5% [103].

This evidence, however, comes from selected patient groups, and some patients received chemotherapy alongside HIFU [103,104]. Whether imaging after treatment can reliably show complete tumour destruction is also unclear. Bielack et al. highlighted this limitation and argued that the evidence was not sufficient to use HIFU instead of surgery for primary bone sarcomas [105]. Emerging data in recurrent or anatomically challenging soft-tissue tumours similarly suggest feasibility, but these applications remain preliminary and should be described as investigational or highly selected [106].

### 6.4. Bone Metastases

The most established musculoskeletal indication for HIFU is pain palliation in painful bone metastases. Bone metastases can cause severe pain, impaired mobility, increased opioid requirements, and reduced quality of life. External-beam radiotherapy remains the standard palliative treatment, but pain relief may be delayed, incomplete, or limited by prior radiation exposure. In this setting, MRgFUS offers a non-invasive local treatment option, particularly for patients with persistent pain after radiotherapy, contraindications to radiotherapy, or who refuse further radiation treatment [87,90].

Pain relief after HIFU is thought to result mainly from heating of the cortical bone surface and periosteal denervation, with additional contributions from local tumour injury and modulation of pain pathways. This mechanism may help explain the relatively rapid onset of analgesia reported in several studies. HIFU may therefore be especially useful when rapid non-invasive pain control is desired and the lesion is technically accessible [107,108].

Early clinical and multicentre studies have established the feasibility of MRgFUS for pain palliation in painful bone metastases, with reported reductions in pain intensity, analgesic or opioid use, and improvements in functional status or quality of life in selected patients [109,110,111]. Liberman et al. followed 25 patients for three months after treatment. Eighteen (72%) had significant pain improvement, and mean VAS scores fell from 5.9 to 1.8. Pain relief was often seen early, with 52% of patients reporting substantial improvement within three days. Among those with available medication data, 67% also reduced their opioid use [111]. Additional studies have expanded this evidence by evaluating imaging response, diffusion and perfusion changes, intraosseous versus extraosseous lesion behaviour, and clinical pain outcomes after treatment [112,113,114]. Although these findings support the palliative value of MRgFUS, the evidence remains limited by small cohorts, heterogeneous patient populations, variable prior treatments, and predominantly non-randomized designs [107,108,115].

Hurwitz et al. later tested MRgFUS against placebo in a phase III trial of 147 patients. At three months, 64.3% of patients treated with MRgFUS met the pain-response endpoint compared with 20.0% in the placebo group (*p* < 0.001). Mean pain scores decreased by 3.6 points with MRgFUS and by 0.7 points with placebo [116]. This trial supports MRgFUS as a non-invasive treatment with randomized controlled evidence for pain palliation in this setting. Subsequent reviews have therefore positioned HIFU primarily as a palliative analgesic technique rather than a general substitute for radiotherapy, surgery, or cement-based stabilization [87,90].

Beyond pain palliation, the available imaging-based evidence suggests that HIFU may produce local tissue effects in some treated bone metastases. However, evidence for durable local tumour control remains less mature than the evidence for analgesic benefit [112,113,114]. Therefore, HIFU should be described cautiously as a technique with potential local tumour effects in selected lesions, while analgesic benefit remains the most robustly supported outcome.

HIFU occupies a distinct role in musculoskeletal interventional oncology. Its best-supported application is pain palliation for technically accessible painful bone metastases, particularly in patients with inadequate response to radiotherapy or limited options for repeat radiation. Its role in osteoid osteoma and desmoid tumours is promising but selective, while its use in primary malignant bone and soft-tissue tumours remains investigational or limited to highly selected cases. Careful patient selection, acoustic targetability, thermal monitoring, and assessment of skeletal stability are essential for safe and effective use.

## 7. Discussion

The expanding role of image-guided interventions has substantially changed the therapeutic landscape of musculoskeletal oncology. Rather than competing with conventional surgery or radiotherapy, these techniques have increasingly become complementary components of multidisciplinary cancer care. Treatment selection is now guided by tumour biology, anatomical location, expected morbidity, patient performance status, skeletal stability, and therapeutic intent [2,4]. Although these techniques are often discussed together, they do not serve identical purposes: thermal ablation primarily targets tumour destruction and biological pain mechanisms, whereas cementoplasty mainly addresses mechanical pain and skeletal instability. Representative clinical evidence, outcomes, and limitations for these techniques are summarized in Table 5.

Radiofrequency ablation has a long history in musculoskeletal tumour treatment and remains widely used in clinical practice. Its role is well established in osteoid osteoma, where it is now the preferred minimally invasive treatment [16]. When either RFA or cryoablation could be used, the choice depends largely on the lesion. RFA is particularly suited to small, well-defined lesions that can be reached safely, with osteoid osteoma being the clearest example [15,16]. Cryoablation can be more useful for larger or irregular lesions, or when the tumour lies close to critical structures because the ice ball can be followed during treatment [20,41].

RFA is also used in metastatic bone disease, most often to relieve pain, although local tumour control can be achieved when the lesion can be fully covered by the ablation zone [30,31,36]. Treating larger or irregular tumours can be more difficult, particularly when they lie close to major nerves or vessels. The ablation zone cannot be seen directly during the procedure, which can make treatment margins harder to judge. Local blood flow may also reduce heating through the heat-sink effect. For some lesions, this makes another ablation technique a more practical choice, even though RFA remains an established and widely available option.

Cryoablation can be useful in some of these situations. The ice ball is visible on imaging as it forms, allowing the operator to follow the treated area during the procedure [41]. This can be helpful near the skin, cartilage, nerves, or the spinal canal, where treatment margins need to be monitored closely. Several cryoprobes can also be used together, giving more flexibility when treating larger or irregularly shaped tumours. Cryoablation has been used for desmoid tumours, recurrent soft-tissue tumours, chordomas, and metastatic lesions. The freezing phase is also generally well tolerated, which can be useful when treatment is performed for palliation. Cryoablation does, however, have practical drawbacks. It is more expensive, procedures may take longer, and the technique is not as widely available as RFA. Current evidence therefore supports cryoablation not as a replacement for RFA, but as a complementary option whose use depends on lesion size, geometry, location, and proximity to critical structures [47,64].

HIFU occupies a different clinical position from percutaneous ablative techniques. Unlike RFA and cryoablation, HIFU offers non-invasive thermal treatment without needle insertion or ionizing radiation exposure. This makes it particularly attractive for selected patients, especially those with technically accessible painful bone metastases or benign lesions in whom avoidance of puncture and radiation is desirable [90]. Prospective studies and randomized evidence support its role in pain palliation for painful bone metastases, while early comparative experience in osteoid osteoma suggests that HIFU may achieve clinical outcomes similar to RFA in selected targetable lesions [96,116]. However, wider adoption remains limited by acoustic window requirements, lesion accessibility, prolonged treatment times, the need for specialized MR-guided platforms, and the absence of mechanical stabilization [93]. For now, its use is best suited to selected patients in whom the lesion can be safely targeted and the advantages of a non-invasive approach are clinically relevant.

Treatment of musculoskeletal tumours involves more than controlling the tumour itself, as skeletal stability also needs to be considered. This is where cementoplasty differs from thermal ablation. RFA, cryoablation, and HIFU mainly target the tumour and biologically mediated pain, whereas cement augmentation is used to reinforce weakened bone and relieve mechanical pain [68,69]. Some patients need both tumour treatment and mechanical support. In these cases, ablation and cementoplasty can be used together during the same procedure. Such combined approaches are particularly relevant in osteolytic metastases involving vertebral bodies, the pelvis, the acetabulum, or other weight-bearing structures, where pain relief alone may be insufficient without restoration of mechanical support [81].

Despite these encouraging developments, several limitations continue to characterize the current evidence. Most available studies are retrospective, include relatively small patient populations, and combine heterogeneous tumour types, anatomical sites, and treatment indications. Procedural protocols, imaging guidance, outcome measures, and follow-up schedules also vary considerably between institutions, making direct comparison across techniques difficult. As a result, many current decisions still rely on multidisciplinary judgment, institutional experience, and lesion-specific technical considerations rather than high-level comparative evidence.

No single technique is suitable for every musculoskeletal tumour. The choice often comes down to the lesion being treated: its biology and location, the ablation margin that can be achieved, and whether the bone also needs mechanical support. Previous treatment and the patient’s goals are equally relevant. Image fusion, robotic navigation, and better thermal monitoring may help with treatment planning and make difficult procedures more precise. Ablation can also be combined with stabilization when tumour control and mechanical support are both needed. Image-guided procedures are already well established for pain relief. Their place in local tumour treatment is less clearly defined and will depend on better clinical evidence.

## 8. Conclusions

Image-guided procedures are now used in several areas of musculoskeletal oncology. RFA is the preferred minimally invasive treatment for osteoid osteoma. RFA, cryoablation, and HIFU are also used to relieve pain from bone metastases, while cementoplasty provides support when tumour involvement has weakened the bone. Thermal ablation has a more selective role in local treatment of limited metastatic disease and in some benign or locally aggressive tumours. Evidence for primary bone and soft-tissue sarcomas is still limited, and these procedures should not be used in place of standard oncologic treatment. The choice of procedure depends largely on the lesion and the reason for treatment. Pain relief, local tumour control, and skeletal stability may each influence this decision, and some patients require more than one of these to be addressed.

## Figures and Tables

**Table 1:** Use of radiofrequency ablation in musculoskeletal oncology: main advantages and limitations across selected clinical scenarios.

Clinical Scenario	Goal of RFA	Advantages	Limitations
Painful bone metastases	Pain palliation	Rapid pain relief, reduces opioid use	Does not restore stability
Spinal metastases	Pain control and local tumour destruction	Minimally invasive, effective in difficult surgical candidates	Risk near neural structures
Oligometastatic disease	Local tumour control	Potentially durable control of limited disease	Careful patient selection required
Osteoid osteoma	Curative treatment	High success rates, low recurrence	Requires accurate nidus localization
Recurrent lesions after radiotherapy	Additional local treatment option	Can be used when radiation options are exhausted	Heat-related complications

**Abbreviations:** RFA, radiofrequency ablation.

**Table 2:** Complications reported with radiofrequency ablation and cryoablation in selected musculoskeletal oncology studies.

Technique	Study	Clinical Setting	Reported Complications	Reported Complication Rate
RFA	Goetz et al. [30]	Painful osteolytic bone metastases, 43 patients	Second-degree skin burn, transient bowel and bladder incontinence, and acetabular fracture	3/43 (7.0%)
RFA	Dupuy et al. [31]	Painful osseous metastases, 55 patients	Grade 3 toxicities: RFA-related pain, neuropathic pain, and foot drop	3/55 (5.4%)
RFA	Levy et al. [32]	Painful bone metastases, 100 patients	Four treatment-related adverse events; two led to hospitalization for pneumonia and respiratory failure	4 adverse events/100 treated patients
RFA	Tanigawa et al. [33]	Painful bone metastases, 33 patients	Grade 3 pain, Grade 2 hypotension, and two Grade 1 burns	4/33 (12.1%)
Cryoablation	Auloge et al. [46]	Primary or metastatic bone tumours, 320 tumours	Fracture, infection, tumour seeding, bleeding, and severe hypotension	29/320 (9.1%)
Cryoablation	Callstrom et al. [47]	Painful bone metastases, 61 patients	Osteomyelitis at the ablation site	1/61 (2%)
Cryoablation	Jennings et al. [48]	Painful bone metastases, 66 patients	Serious procedure- or device-related/possibly related adverse events	3/66 (4.5%)
Cryoablation	Pal et al. [49]	Recurrent or metastatic soft-tissue sarcoma, 141 patients; 217 procedures	Two bleeding episodes requiring embolization, transient saphenous nerve injury, and pneumothorax	4/217 procedures (2%)

**Abbreviations:** RFA, radiofrequency ablation.

**Table 3:** Use of cryoablation in musculoskeletal oncology: main advantages and limitations across selected clinical scenarios.

Clinical Scenario	Goal of Cryoablation	Advantages	Limitations
Painful bone metastases	Pain palliation	Rapid and durable pain relief	Fracture risk in large lytic lesions
Spinal metastases	Local tumour control and pain reduction	Real-time visualization of the ice ball	Requires thermal protection of neural structures
Pelvic and acetabular metastases	Palliation and stabilization planning	Large ablation volumes possible	Often requires adjunctive cementoplasty
Oligometastatic disease	Local tumour control	Potential for durable local control in selected patients	Requires complete ice-ball coverage and adequate margins
Recurrent disease after radiotherapy	Salvage treatment	Can be repeated and combined with other therapies	Limited evidence in some tumour types

**Table 4:** Use of cementoplasty in musculoskeletal oncology: main indications, expected benefits, limitations, and adjunctive procedures across selected anatomical sites.

Location	Main Indication	Expected Benefit	Limitations	Adjunctive Procedures
Vertebra	Painful metastatic compression fractures, instability	Rapid pain relief, vertebral stabilization	Cement leakage, epidural extension	RFA, MWA, cryoablation, kyphoplasty
Sacrum	Painful sacral metastases or insufficiency fractures	Pain reduction, improved sitting and ambulation	Complex anatomy, neural proximity	Sacroplasty, thermal ablation
Acetabulum	Osteolytic destruction causing weight-bearing pain	Improved gait and load transmission	Extensive cortical loss	Ablation, screw fixation
Pelvis	Painful osteolytic metastases	Mechanical stabilization and pain relief	Irregular anatomy	Ablation, osteosynthesis
Long bones	Selected painful lytic lesions or impending fracture risk	Pain relief and limited reinforcement	Limited resistance to torsional forces	Screw fixation, osteosynthesis

**Abbreviations:** RFA, radiofrequency ablation; MWA, microwave ablation.

**Table 5:** Representative clinical evidence, outcomes, and limitations of interventional treatments for musculoskeletal tumours.

Technique	Clinical Setting	Representative Evidence	Main Findings	Main Limitations/Patient Selection	Key Reference(s)
**RFA**	Osteoid osteoma	Woertler et al., 47 patients; Cioni et al., 38 patients; clinical series	Primary clinical success was 94% in Woertler et al., reaching 100% after repeat treatment. Cioni et al. reported 78.9% primary and 97.2% secondary success, with a mean follow-up of 35.5 months.	Accurate nidus localization is required. Lesions close to nerves, cartilage, or skin need careful thermal protection.	[23,24]
**RFA**	Selected intermediate and large bone tumours	Nakatsuka et al.: Phase II study, 20 patients	Tumour response was seen in 16/18 evaluable patients (88.9%), and pain decreased by ≥2 points in 11/13 (84.6%).	Small, selected cohort. Larger treatment volumes can be difficult to cover safely, particularly near critical structures.	[19]
**Cryoablation**	Osteoid osteoma	Le Corroller et al.: retrospective single-centre study, 50 patients	Clinical success was 48/50 (96%). Median VAS fell from 8 to 0 at 6 weeks and remained 0 at longer follow-up. One recurrence was successfully retreated.	RFA has the longer-established evidence base. Cryoablation may be useful when visualization of the ice ball is particularly helpful.	[52]
**Cryoablation**	Progressive desmoid tumours	CRYODESMO-01: prospective multicentre study, 50 patients	At 12 months, 86% of patients had no disease progression, with improvement in pain and function.	Many desmoid tumours can initially be observed. Treatment is mainly considered for progressive, symptomatic, or function-threatening disease.	[55,98]
**Cryoablation**	Oligometastatic musculoskeletal disease	McMenomy et al.: retrospective series, 40 patients, 52 lesions	Local control was achieved in 45/52 lesions (87%) at a median follow-up of 21 months.	Results come from a selected group with limited metastatic disease and should not be applied to widespread metastases.	[62]
**Cementoplasty**	Painful malignant vertebral lesions	Weill et al., Fourney et al.: clinical series	Weill et al. reported pain improvement after 31/33 evaluable procedures, with benefit maintained in 73% at 6 months. Fourney et al. reported marked or complete pain relief in 84% of evaluable procedures, with significant pain reduction up to 1 year.	Careful selection is needed in patients with posterior wall destruction, epidural extension, or major instability. Cement leakage may occur.	[75,77]
**Cementoplasty**	Painful extraspinal osteolytic lesions	Botton et al., Anselmetti et al.: clinical series	Botton et al. reported complete or partial pain relief after 88% of procedures. Anselmetti et al. reported marked pain reduction and suspension of narcotic drugs in 47/50 patients.	Cement does not provide tumour control and may be insufficient for long-bone lesions exposed to bending and torsional forces. Fixation may be required.	[72,79]
**HIFU**	Painful bone metastases	Hurwitz et al.: Phase III randomized placebo-controlled trial, 147 patients	Pain response at 3 months was 64.3% with HIFU vs. 20.0% with placebo (*p* < 0.001).	A suitable acoustic window is required. HIFU does not provide mechanical stabilization.	[116]
**HIFU**	Osteoid osteoma	Masciocchi et al.: propensity-matched observational study; Sharma et al.: small comparative study	Masciocchi et al. reported success in 14/15 (93.3%) HIFU patients and 15/15 RFA patients. Sharma et al. reported 8/9 HIFU and 9/9 RFA patients were pain-free at 4 weeks.	Evidence is based on small, selected groups. Lesions close to skin, major nerves, the physis, or without a safe acoustic pathway may not be suitable.	[96,97]
**HIFU**	Recurrent or unresectable malignant bone tumours	Yu et al.: retrospective study, 27 patients with recurrent unresectable osteosarcoma	Response rate was 51.8%, local disease control was 85.2%, median local progression-free survival was 14 months, and median overall survival was 21 months.	Evidence remains limited and non-randomized. Imaging response should not be considered equivalent to complete surgical resection, and HIFU should not replace standard sarcoma treatment.	[102,105]

**Abbreviations:** HIFU, high-intensity focused ultrasound; RFA, radiofrequency ablation; VAS, visual analogue scale. Note: Results are presented as reported in the individual studies and are not directly comparable because of differences in study populations, treatment indications, outcome definitions, and follow-up durations.

## Data Availability

No new data were generated or analyzed in this study. Data sharing is not applicable to this article.
